# CTLA-4 checkpoint blockade in breast cancer, a case in point report

**DOI:** 10.1186/2051-1426-3-S1-O4

**Published:** 2015-08-14

**Authors:** Chiara Dellepiane, Michela Lia, Mario Roberto Sertoli

**Affiliations:** 1 Clinic of Medical Oncology, IRCCS SAN MARTINO IST, Genoa University, Genoa, Italy; 2 EORTC, Bruxelles, Belgium

## 

A patient concomitantly affected by breast cancer and melanoma is presented in order to contribute to the ongoing debate on breast cancer immunotherapy [1,2]. In 1996 the patient was operated on for left early breast cancer and treated with adjuvant radiotherapy and 5 years Tamoxifen. In 2009 biochemical progression and bone metastases appeared so Letrozole hormonal therapy was carried out, ensuing in clinical stabilization. In 2010, a left arm cutaneous melanoma was excised (pT4a Breslow 4.5 mm), and axillary nodal dissection detecting an involved node. Because of subsequent in-transit metastases, the patient underwent chemo-hyperthermic perfusion, reaching a dermic complete response (CR). Suddenly melanoma progressed in the lung and liver, as confirmed by ultrasound driven biopsy. Chemotherapy (DTIC) was given, achieving visceral partial response. At this point Ipilimumab [3] was available and CR of liver, lung, and dermic metastases was obtained after 4 cycles, while bone lesions remained stable and considered breast cancer. Four months later novel liver metastases appeared and biopsy unexpectedly confirmed their breast cancer origin. The patient after unsuccessful chemotherapy, finally died of liver failure, without evidence of melanoma metastases. This very special case shows the impressive discrepancy in response to CTLA4 check-point therapy between melanoma and breast cancer.
